# A pan-influenza monoclonal antibody neutralizes H5 strains and prophylactically protects through intranasal administration

**DOI:** 10.1038/s41598-024-53049-5

**Published:** 2024-02-15

**Authors:** Anna L. Beukenhorst, Jacopo Frallicciardi, Keira L. Rice, Martin H. Koldijk, Joana C. Moreira de Mello, Jaco M. Klap, Christoforos Hadjichrysanthou, Clarissa M. Koch, Kelly A. S. da Costa, Nigel Temperton, Babette A. de Jong, Helene Vietsch, Bertjan Ziere, Boris Julg, Wouter Koudstaal, Jaap Goudsmit

**Affiliations:** 1 Department of Biostatistics, Harvard T.H. Chan School of Public Health, Boston, MA USA; 2 Leyden Laboratories BV, Leiden, The Netherlands; 3 Centre for Epidemiology, University of Manchester, Manchester Academic Health Science Centre, Manchester, UK; 4 Department of Mathematics, University of Sussex, Brighton, UK; 5 Viral Pseudotype Unit, Medway School of Pharmacy, University of Kent and University of Greenwich, Chatham, UK; 6 Departments of Epidemiology, Immunology and Infectious Diseases, Harvard TH Chan School of Public Health, Boston, MA USA

**Keywords:** Antibody therapy, Influenza virus, Viral infection, Public health, Drug development, Translational research

## Abstract

Avian A(H5N1) influenza virus poses an elevated zoonotic threat to humans, and no pharmacological products are currently registered for fast-acting pre-exposure protection in case of spillover leading to a pandemic. Here, we show that an epitope on the stem domain of H5 hemagglutinin is highly conserved and that the human monoclonal antibody CR9114, targeting that epitope, potently neutralizes all pseudotyped H5 viruses tested, even in the rare case of substitutions in its epitope. Further, intranasal administration of CR9114 fully protects mice against A(H5N1) infection at low dosages, irrespective of pre-existing immunity conferred by the quadrivalent seasonal influenza vaccine. These data provide a proof-of-concept for broad, pre-exposure protection against a potential future pandemic using the intranasal administration route. Studies in humans should assess if autonomous administration of a broadly-neutralizing monoclonal antibody is safe and effective and can thus contribute to pandemic preparedness.

## Introduction

Highly pathogenic avian influenza virus poses an ‘elevated zoonotic threat’ to humans, especially A(H5N1), given its recent unprecedented spread among wild birds, poultry, and mammals in 67 countries on five continents^1,2^. Since 2020, A(H5N1) has become a year-round rather than a seasonal infection, resulting in a dramatic increase in poultry losses due to death, measurable declines in wildlife bird populations in many regions of the world, and culling (~ 190 M in 2021–2023 alone, compared to ~ 195 M in 2005–2019)^3^. The World Health Organization has reported 878 cases of human infection with A(H5N1) since 2003, of which 458 were fatal (case fatality rate of 52%)^4^. However, to date the basic reproductive number R_0_ in these outbreaks has been below unity in value and hence they have not triggered epidemics in humans. The threat to humans has been growing, however: spillover into mammals has led to mammalian adaptation and resulted in mutations previously shown to enable airborne transmission between ferrets in gain-of-function experiments (e.g. PB2 E67K)^5–7^.

No fast-acting prophylactics are available to prevent infection with a future A(H5N1) strain that could be transmitted between humans. Some existing antiviral drugs for seasonal influenza cross-protect against A(H5N1), but these are mostly suitable as post-exposure treatments and concomitant risk of resistance from viral escape mutants^8,9^. Post-exposure transfer of convalescent serum is effective, but supply constraints and cost make this an unsuitable treatment on a larger scale^10^. Some governments have a stockpile of H5 vaccines (US—vaccine doses for 20 million people^11,12^) or advance purchase agreements (UK—100 million vaccine doses^13^). However, these vaccines may not work if another H5 subtype (e.g. H5N2, H5N8) spills over to humans. Furthermore, the recent SARS-CoV-2 pandemic showed that vaccination, although highly important to avert severe disease, does not necessarily prevent infection and associated spread^14^, especially when vaccine roll-out is still ongoing and before the vaccine has elicited antibody responses in a sufficient proportion of the population. Therapeutic monoclonal antibodies have saved lives of infected patients during the SARS-CoV-2 pandemic, but no prophylactic monoclonal antibodies have been approved by the Food and Drug Administration (FDA) or European Medicines Agency (EMA).

Prophylactic monoclonal antibodies could prevent community spread in the period before and during vaccination campaigns, but pre-exposure administration of anti-influenza antibodies through the systemic route has failed in human trials^15^, with VIR-2482 as latest example. Currently, it is unknown whether passive immunization with antibodies delivered into the nasal cavity, at the port of entry for influenza virus, could slow down a pandemic in the period before and during vaccine roll-out.

Here, we assess in vitro breadth of a broadly neutralizing human monoclonal antibody, CR9114, against a range of influenza A(H5) strains from various subtypes. We report intranasal prophylactic protection by CR9114 against lethal H5N1 challenge in mice with and without pre-existing immunity conferred by the quadrivalent seasonal influenza vaccine.

## Results

### Potency and breadth of human monoclonal IgG1 antibody CR9114

**Figure 1:**
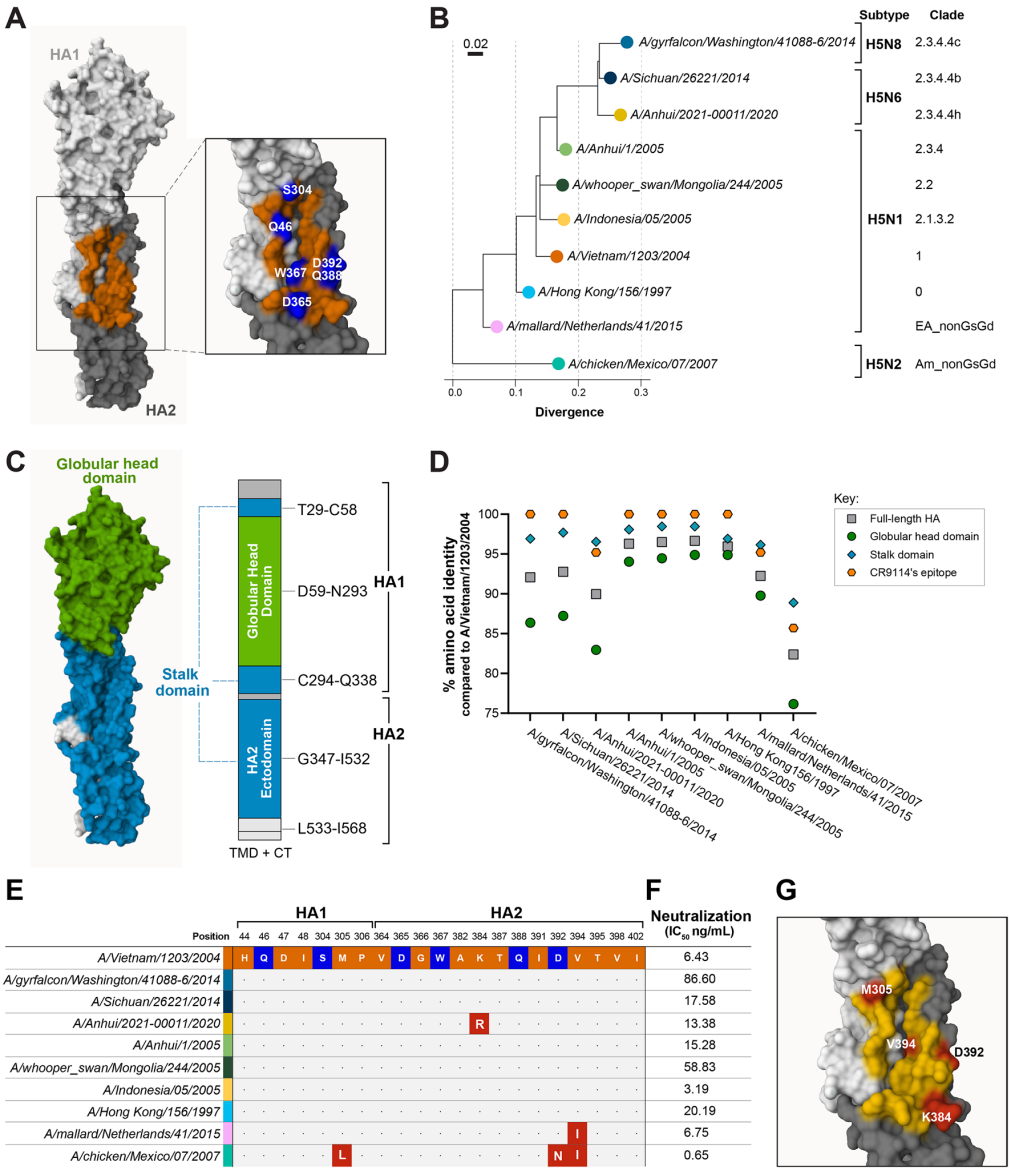
CR9114 neutralizes H5 strains belonging to different clades in vitro due to the high level of conservation of its epitope, and even in case of epitope substitutions. (**A**) CR9114’s epitope on A/Vietnam/1203/2004 HA^16^. The surfaces of HA1 and HA2 subunits are shown in white and grey, respectively. Residues in the epitope are shown in orange, while hotspots, defined as residues forming H-bonds with CR9114, are shown in blue**.** The numbering of the residues follows the amino acid sequence alignment shown in Supplementary Fig. S1. (**B**) Phylogenetic tree based on the identity of the hemagglutinin protein sequence, showing the divergence among the strains used in the pseudotype virus neutralization assay (PNA). The selected strains belong to different clades. A/chicken/Scotland/1959 (not shown) was used as a root for building the tree. The full tree is presented in Supplementary Fig. S2. The list of details regarding the H5 strains is reported the Supplementary Table S1. (**C**) Schematic depiction of the full-length hemagglutinin from A/Vietnam/1203/2004 HA showing the boundaries of the globular head and of the stalk domains. The stalk domain consists of the N- and C-terminal parts of HA1 subunit, and the HA2 ectodomain; the transmembrane domain (TMD), and the cytoplasmic tail (CT) on the HA2 are not shown on the 3D structure. (**D**) Percent identity of amino acid sequence of the strains tested in the PNA compared to the full-length HA, globular head domain, stalk domain, and CR9114’s epitope of A/Vietnam/1203/2004. (**E**) Sequence alignment of CR9114’s epitope on H5 HA from A/Vietnam/1203/2004 and the other strains used in the PNA. Hot spots are highlighted in blue. (**F**) Neutralization values as IC_50_ values (ng/mL) generated by PNA of CR9114 against ten H5 strains belonging to difference clades. (**G**) CR9114’s epitope on the HA of the strains with amino acid substitutions compared to A/Vietnam/1203/2004 tested in the PNA. Residues in yellow are fully conserved, whereas residues in red have been substituted in at least one of the tested strains. Figure 1A, C and G have been generated in Mol* Viewer^19^ with PDB ID: 4FQI. Single-letter abbreviations for amino acids: *A* Ala, *D* Asp, *G* Gly, *H* His, *I* Ile, *K* Lys, *L* Leu, *M* Met, *N* Asn, *P* Pro, *Q* Gln, *S* Ser, *T* Thr, *V* Val, *W* Trp.

CR9114 is a human monoclonal IgG1 antibody that targets an epitope on the hemagglutinin (HA) stalk domain of influenza A1, A2 and B^16^. Its flexible paratope enables binding to all influenza A subtypes and both influenza B lineages^17^. Previous research has suggested that CR9114 is a product of sequential exposure of its donor to influenza H1N1, H3N2 and then influenza B^18^. A crystal structure of the CR9114-H5N1 hemagglutinin (HA) complex with epitope-to-paratope interactions is available (PDB ID: 4FQI). The epitope of CR9114 on the HA of A/Vietnam/1203/2004 consists of 7 residues in HA1 subunit and 14 in the HA2 subunit, with 6 residues (2 in the HA1 and 4 in the HA2) forming H-bonds with the antibody (Fig. 1A)^16^.

We tested CR9114’s neutralization potency against phylogenetically distant influenza H5 strains. We selected ten strains isolated between 1997 and 2020, from diverse subtypes (H5N1, H5N2, H5N6, and H5N8) and clades (0, 1, 2.1.3.2, 2.2, 2.3.4.4, 2.3.4.4b, 2.3.4.4c, 2.3.4.4 h, Am_nonGsGD, and EA-nonGsGD), including A/Vietnam/1203/2004 as a reference strain (Fig. 1B, Supplementary Table S1). Protein sequence alignment of the tested strains against the A/Vietnam/1203/2004 HA reveals the high level of conservation of CR9114’s epitope on H5 HA (Fig. 1C,D). In seven strains, the sequence identity is 100% compared to the reference strain. Two strains (clades 2.3.4.4 h and EA_nonGsGD) have cumulated one amino acid substitution, and one strain (clade Am_nonGsGD) has cumulated more than one substitution (Fig. 1E,G). The two most phylogenetically distant strains (clades Am_nonGsGD and EA_nonGsGD) share the same substitution (V394I). Overall, the amino acid sequence of the CR9114’s epitope (and, in general, the stalk domain) is far more conserved than the full-length HA and the globular head domain, even in the strains that cumulated substitutions. The globular head domain always shows the highest genetic variability between strains.

CR9114 potently neutralizes all tested strains with IC_50_ values in the range of 0.646–86.6 ng/mL (Fig. 1F), including H5 strains with other neuraminidase subtypes than N1 (N2, N6, N8) or from other clades than clade 1. The most potently neutralized strain, A/chicken/Mexico/07/2007 (IC_50_ = 0.646 ng/mL), carries three mutations compared to A/Vietnam/1203/2004. The three mutated residues, L305, N392 and L394, are also common in H1N1 strains. Both other strains with epitope mutations compared to A/Vietnam/1203/2004 are also potently neutralized (IC_50_ = 6.75 ng/mL and 13.38 ng/mL). The least potently neutralized strain is also phylogenetically furthest removed from A/chicken/Mexico/07/2007.

These data suggest that phylogenetic distance from A/chicken/Mexico/07/2007 is a bigger factor in neutralizing potency of CR9114 than phylogenetic distance from A/Vietnam/1203/2004, or substitutions in the epitope compared to A/Vietnam/1203/2004. Although sequence identity of the CR9114 epitope on A/chicken/Mexico/07/2007 is lower compared to that on A/Vietnam/1203/2004 (85.71%), it has the highest sequence identity with the epitope on the consensus H1N1 strain (76.2%) compared to the other strains (A/mallard/Netherlands/41/2015: 66.7%; all other strains: 61.9%; see Supplementary Table S3).

### Intranasally-delivered CR9114 protects mice against lethal A(H5N1) challenge at low dosage, with and without pre-existing immunity

We compared pre-exposure efficacy of intranasally-administered CR9114 against A(H5N1) both in naïve mice and mice immunized with seasonal influenza. The mice that received quadrivalent Influvac® seasonal flu vaccine (*N* = 30) had high hemagglutinin inhibition titers against A(H1), A(H3), B/Yam and B/Vic (range 28–320; Supplementary Table S2), whereas the mice injected with phosphate-buffered saline (PBS; *N* = 30) had titers below lower limit of detection.

**Figure 2:**
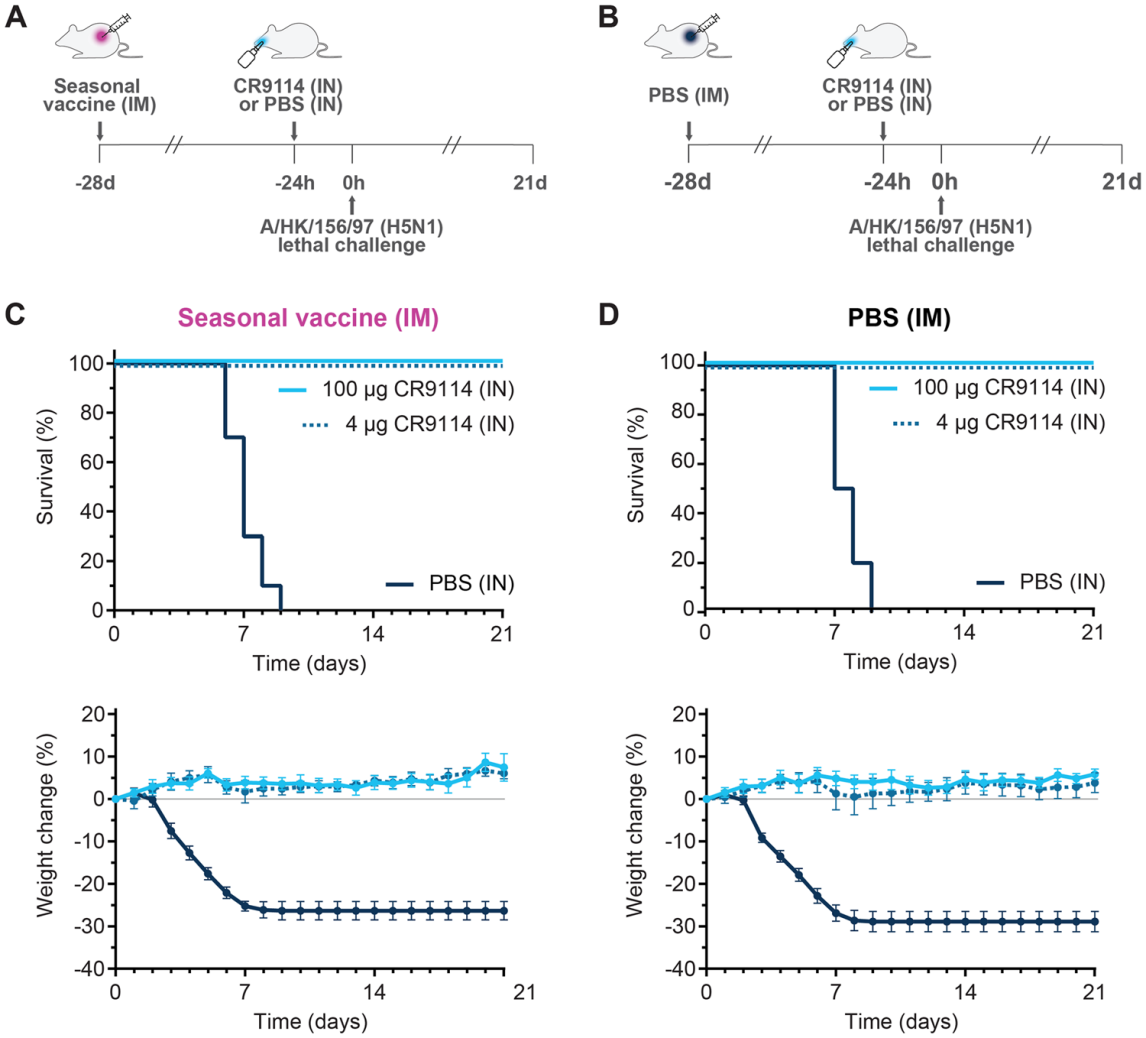
Prophylactic intranasal administration of CR9114 protects mice against A(H5N1), both in the absence and in the presence of pre-existing immunity elicited by the seasonal influenza vaccine. Schematic depiction of the study design testing the prophylactic efficacy of intranasally administered CR9114 versus PBS (-24 h) in mice immunized intramuscularly with Influvac® quadrivalent vaccine (**A**), or PBS at 28 days before challenge (**B**). Percentages of survival (*top panels*) and weight change (*bottom panels*) of mice challenged with A/HK/156/97 (H5N1) 24 h after intranasal administration with 4 µg CR9114 (0.2 mg/kg assuming each mouse weights 20 g), 100 µg CR9114 (5 mg/kg assuming each mouse weights 20 g) or PBS in the (**C**) presence of pre-existing immunity from intramuscular Influvac® quadrivalent vaccine, and (**D**) absence of pre-existing immunity (intramuscular PBS). n = 10 per group, except the vaccinated group receiving 4 µg CR9114, where n = 9. *IM* intramuscular, *IN* intranasal.

Intranasal CR9114 (high dosage 100 µg—5 mg/kg assuming each mouse weights 20 g—or low dosage 4 µg—0.2 mg/kg) or PBS were administered 24 h before viral challenge (Fig. 2A,B). Mice that received intranasal PBS all died within 9 days, irrespective of seasonal vaccination status. CR9114 protected 100% of mice from mortality and weight loss even at the low dosage of 4 µg, both in the presence and absence of pre-existing immunity conferred by the seasonal vaccine (Fig. 2C,D).

## Discussion

In this study, we have shown that pre-exposure passive immunization with the broadly-neutralizing antibody CR9114 via the intranasal route fully protects mice against lethal A(H5N1) challenge irrespective of the presence or absence of immunity against seasonal influenza while, as expected, the seasonal vaccine did not. An in vivo proof-of-concept is a prerequisite for clinical development, but studies into safety and efficacy are needed to extend our findings to humans.

If—or when^20^—A(H5N1) acquires the capacity for human-to-human transmission, the virus encounters populations lacking strong pre-existing immunity against H5. In our study, mice were not protected by the quadrivalent seasonal influenza vaccine, even though it contains H1 antigen, which belongs, like H5, to influenza group A1. In humans, it has also been shown that seasonal vaccination does not lead to in vitro heterosubtypic protection: the trivalent seasonal vaccine leads to increased serum titers against H1N1 but not H5N1^21^. An in vivo study with human-to-mouse serum transfer showed that serum of a minority of people (9/25) cross-protects against H5N1, but only shortly after seasonal influenza vaccination. For 9 out of 25 vaccinated individuals, transferred post-vaccination serum protected mice against lethal H5N1 challenge. The authors showed that protection was transient, and not boosted by subsequent repeated vaccination^22^. Of note, some people may be cross-protected against H5N1 because of high serum titers against N1, elicited by prior infection to H1N1, or prior vaccination^23^.

It is difficult to prepare for a pandemic, as it is unknown which zoonotic threats spillover to humans. Tools for pandemic preparedness toolbox should therefore be broad, and prepare against a range of known and unknown viruses. We showed that the CR9114 epitope is conserved on H5 strains from different clades and that CR9114 potently neutralizes H5 pseudotypes expressing N1, N2, N6, and N8. The most potently neutralized H5N1 strain has the highest sequence identity in the CR9114 epitope with H1N1 viruses. However, even viruses with different residues in the CR9114 epitope are potently neutralized by CR9114. This is in line with previous research, which showed that CR9114 can tolerate most of the mutations in the 21 residues of its epitope, as it binds strains with a variety of point mutations^16^.

These results suggest a high tolerability of CR9114 to variation in influenza H5 strains. It is not possible to investigate the limits to tolerability of variation in influenza H5 strains in escape studies, as such ‘gain-of-function’ experiments are highly regulated because of the risks for creating highly pathogenic strains. However, escape of H1N1 strains from CR9114 has been investigated. No H1N1 escape mutants have been found after in vitro passaging of H1N1 under pressure of CR9114^24^. CR9114 retained binding ability, sufficient neutralization potency and Fc and FcR-mediated effector functions to the resulting virus (in contrast to other antibodies tested). Another study tested all possible amino acid substitutions in on three H1N1 strains, in the 8 residues that are involved in binding to CR9114 (~ 150 viral variants with single mutations; ~ 10,000 viral variants with double mutations). None of these substitutions affected the ability of CR9114 to bind, neutralize or protect against these strains.

We tested CR9114 neutralizing activity on pseudotyped viruses rather than live virus. Pseudotypes viruses do not produce replication-competent progeny and therefore do not require high biosafety laboratories for handling^25^. Meta-analyses have shown that pseudovirus neutralization highly correlates with live virus neutralization. Moreover, pseudovirus neutralization assays are highly sensitive, although pseudotyped virus may differ in distribution of hemagglutinin and neuraminidase glycoproteins on the virion compared to live virus^26^.

As CR9114 also potently inhibits other HA and NA subtypes, including H3, H7, N7 and N8, and protects against influenza A and B strains, the findings reported herein may also generalize to other zoonotic threats, such as H5N8, H3N8 and H7N7^17^. It also neutralizes influenza subtypes like H17 and H18, which had not yet been discovered when the antibody CR9114 was found^17^. Broadly-neutralizing antibodies like CR9114, that protect against all known subtypes of a particular viral family, could therefore constitute a broad tool for pandemic preparedness. The SARS-CoV-2 pandemic has shown that, if needed, antibodies against pandemic viruses can be developed relatively fast—this was a worldwide, billion-dollar effort resulting in various antibodies approved by the FDA and EMA through emergency authorization pathways. Retraction of emergency authorization was also fast when new variants escaped binding by antibodies that targeted areas on the SARS-CoV-2 spike protein that were highly mutable. Most of the therapeutic antibodies targeted such highly mutable areas on the spike, and did not retain efficacy with delta and omicron variants^14^. Currently, only two therapeutic antibody products (bebtelovimab and combination therapy tixagevimab-cilgavimab) still neutralize some of the circulating omicron variants in vitro^27^.

Intranasal prophylaxis using an antibody like CR9114 may allow for short-term control because it can be autonomously administered and provides immediate protection, possibly against infection and transmission as well as from severe disease^28–30^. This can be especially useful during vaccine roll-out: avian influenza HAs like H5 are less immunogenic than human influenza HAs (or SARS-CoV-2), and therefore require multiple dosages and longer time until immunity is elicited^31^. Once sufficient people have been immunized, vaccination could provide the advantage of durable protection against severe disease. Intranasal antibody sprays could still be used for the immunocompromised and elderly, who tend to have poorer immune responses to vaccination, whereas intranasal antibody prophylaxis is equally effective across all age groups. Furthermore, local outbreaks or superspreading events with high risk of infection could be prevented or curbed with intranasal antibody prophylaxis^32^.

We showed prophylactic efficacy of CR9114 administered intranasally, 24 h before challenge. The maximum window of protection is probably longer. One study showed that 1 mg/kg intranasal CR6261, another V_H_1-69 antibody which has 86% sequence identity with the heavy chain of CR9114, protects from 168 h (7 days) prior to lethal H1N1 challenge up to 24 h post-infection^30^. Intranasal administration of CR6261 was still effective against H1N1 at 5, 6 and 7 days before challenge, whereas systemic administration (intraperitoneal injection) was not, suggesting an advantage of intranasal administration. Other antibodies also had windows of protection from at least 168–120 h pre-exposure (4–7 days) to at least 24 h post-exposure^28–30^.

In a real-world pandemic scenario, the frequency of intranasal prophylaxis administration is contingent on the duration of the window of protection. Despite clearance mechanisms of the intranasal mucosa^33,34^, the window of protection in mice is multiple days, even at low dosages^28,29^. Clinical trials in humans, investigating intranasal antibody pharmacokinetics, are needed to guide dose finding, prophylactic antibody thresholds, and investigate corresponding therapy adherence^35^.

From the recent SARS-CoV-2 outbreak we learned that early intervention aiming at limiting human-to-human virus transmission is key in preventing pandemic growth, widespread lockdowns, hospitalizations, and mortality. Innovations for immediate protection in the early phase of human-to-human transmission of emerging viral infections are needed; if available, intranasally administered antibodies that block infection at the port of viral entry may have a crucial role in providing early protection against an infection with H5N1 influenza virus. Collaboration between professionals in public health, immunology, virology, and clinical development will be vital to fill the gap in the pandemic preparedness toolbox in time for the next threat.

## Material and methods (online)

### Challenge study

A challenge study in the murine model was performed to evaluate the impact of vaccine-induced immunity to seasonal influenza against influenza A/HK/156/97 (H5N1) infection and the prophylactic efficacy of intranasal administration of CR9114 against influenza A/HK/156/97.

Here the primary objective was defined as a significant improvement in survival with the vaccinated and treated with CR9114 groups in comparison to the vaccinated control group and the not vaccinated and treated with CR9114 groups in comparison to the not vaccinated control group. The secondary objective was defined as a significant reduction in bodyweight loss for the same comparisons.

With 10 animals per group, the study was powered to have at least 80% power to detect a 58% difference in survival between the control group and the treatment groups, assuming 0% survival in the vehicle control group (2-sided Fisher’s exact test at a 5% significance level, R package statmod version 1.4.36).

During the acclimatization period, all mice (n = 60) remained healthy and were considered suitable for enrolment into the study on the day of vaccination (study day -28). There were no abnormal health observations during the period between vaccination and challenge of the animals. All mice anesthetized prior to CR9114 administration on study day -1 recovered well from the anesthesia. One mouse (vaccinated, to be treated with 4 µg CR9114) died during anesthesia on study day 0. Therefore, this mouse was excluded from statistical analysis.

Six to seven weeks old female BALB/c mice (average weight 20 mg) were assigned to treatment groups using a digital randomizer (n = 10 per group, except the vaccinated group receiving 4 µg CR9114, where n = 9) received an intramuscular dose of 3 μg HA antigen per influenza strain of the seasonal influenza Influvac® quadrivalent vaccine or PBS 28 days before virus challenge. The four vaccine strains were: A/Victoria/2570/2019 (H1N1), A/Cambodia/e0826360/2020 (H3N2), B/Washington/02/2019 (B/Victoria) and B/Phuket/3073/2013 (B/Yamagata).

The personnel involved in dose administration and/or monitoring of the animals were blinded for the treatment received by the animals, as vials containing the test item or placebo were encoded with the group number without any further information on the actual content of the vials.

One day before virus challenge, blood samples were obtained to assess HI antibody titers elicited 4 weeks post immunization. Following blood sampling, the mice received an intranasal dose of 100 µL CR9114 (100 or 4 µg) or PBS. Two PBS control groups were included, one for the vaccinated and one for the PBS control arm. Before intranasal administration of the virus, CR9114 or PBS, the mice were anesthetized with a mixture of 200 µL ketamine-xylazine by intraperitoneal injection. On study day 0 mice were challenged with 50 µL of approximately 12.5 LD_50_ of A/HK/156/97 influenza virus by intranasal inoculation.

In both study arms, mice were monitored daily for survival and body weight loss from study day –1 until the end of the study at day 21. Mice were declared moribund and euthanized immediately when they displayed any of the following signs: rough coat, rolled up position, labored breathing, inactivity in response to manipulation/handlings. At the end of the study on day 21, or earlier when deemed necessary (humane endpoint), mice were euthanized by cervical dislocation.

Serum sample analysis was performed to determine HI titers in pooled serum samples from mice either vaccinated or not with seasonal influenza vaccine. The two pools generated were measured using a hemagglutination inhibition assay for the detection of influenza specific antibodies against the viruses in the Influvac® vaccine. Following performance of the HI assay, results were calculated and expressed as geometric mean HI titers with a LOD of 5. All negative samples were given a value of ≤ 5.

Survival proportions after viral challenge were analyzed using a 2-sided Fisher’s exact test and change in body weight was analyzed using an Area Under the Curve (AUC) analysis. If a mouse was found dead or euthanized during the study, the mouse was weighed, and this measurement was carried forward until the end of the study. The weight per mouse at day 0 was used as baseline and weight change was determined relative to baseline. The net AUCs were compared using a Welch t-test. Comparisons to the control (PBS) group were performed using a stepwise approach within each vaccination arm starting with the highest antibody dose conditionally testing the lower dose if the previous step was statistically significant. Statistical analysis was performed using R (v4.1.0) and statistical significance was set at α = 0.05.

All animal procedures were performed in accordance with the protocols and reviewed and approved by the Dutch Central Authority for Scientific Procedures on Animals.

### Phylogenetic tree and protein sequence alignment

Amino acid fasta files for HA gene from 754 sequences sampled between December 1959 and December 2020 were downloaded from GISAID EpiFlu as well as the respective metadata file up to October 10, 2023 (https://gisaid.org/). The detailed list is available in the accompanying acknowledgement table and in the Supplementary Table S1 for the strains tested in the pseudotype virus neutralization assay. The HA aminoacid sequences for A/Anhui/2021_00011/2020|H5N6 (EPI1848299), A/Sichuan/26221/2014 (EPI533583), and A/Hong_Kong/156/1997 (EPI1328382) were collected by GISAID. The HA sequences for A/Vietnam/1204/2004 (EF541404), A/gyrfalcon/Washington/41088_6/2014 (KP307984), A/Indonesia/5/2005 (CY116646), A/mallard_duck/Netherlands/41/2015 (MF694083), A/chicken/Mexico/07/2007 (KJ729343), A/Anhui/1/2005 (DQ371928), and A/whooper_swan/Mongolia/244/2005 (EU723707) were collected by IRD.

The phylogenetic tree was built based on the Nextstrain workflow for influenza A/H5NX virus evolution with minor changes (available at https://github.com/nextstrain/avian-flu and downloaded on October 12, 2023). A/chicken/Scotland/1959 (the oldest dated strain GISAID, clade EA_nonGsGD) was used as a root for building the tree. After processing and quality filtering, 632 genomes were retained including the 10 tested ones. Tree visualization and plotting were done with ggtree^36^ and treeio^37^ R packages. R version 4.2.2 (2022–10-31). Platform: aarch64-apple-darwin20 (64-bit). Running under: macOS 14.0. The HA amino acid sequences have been aligned using Clustal X with standard settings.

### Breadth of neutralization

The pseudotype neutralization assay was performed following the protocol by Ferrara et al. published in *Methods Protoc*^38^*.* with the Influenza A/H5 strains listed in Supplementary table S1. CR9114 concentrations used were in the range of 0.49–1000 ng/mL. As described in the protocol, fifty microliters of PV at a titer of 1.0 × 10^6^ RLU/well as determined via titration was then added to the mAb. This mixture was incubated for 1 h at 37 ◦C, 5% CO_2_. Afterwards, 50 µL of 1.5 × 10^4^ HEK293T/17 cells were added to each well. PV only (equivalent to 0% neutralization) and cell only controls with no virus (equivalent to 100% neutralization control) were also included in the test plate. Plates were incubated for 48 h at 37 ◦C and 5% CO_2_. Media was removed and 25 µL of the Bright-Glo® luciferase assay substrate added to each well. Plates were then read using the GloMax® Navigator (Promega, Southampton, UK) using the Promega GloMax® Luminescence Quick-Read protocol.

Relative Light Units (RLU) values were normalized relative to the PV only and cells only, where PV only and cell only represent respectively a 0% and 100% neutralization response. The resulting neutralization-antibody dose response curve was modelled using a 4-parameter logistic curve with fixed plateaus at 0 and 100% and a sample specific slope and IC_50_. The IC_50_ values presented in Fig. 1F are the geometric mean of the IC_50_ values calculated for one, two, or three independent experiments.

### Ethical approval statement

All animal procedures were performed in accordance with the protocols and reviewed and approved by the Dutch Central Authority for Scientific Procedures on Animals. The study is reported in accordance with the ARRIVE guidelines.

### Supplementary Information

Supplementary Information.

## Data Availability

The structure of CR9114 in complex with H5 is freely available from the Protein Data Bank (PDB ID: 4FQI; https://www.rcsb.org/structure/4FQI). The sequences from neutralized H5 strains are freely available from GISAID (https://gisaid.org/ with isolate IDs EPI_ISL_244540, EPI_ISL_328966, EPI_ISL_954, EPI_ISL_10656749, EPI_ISL_5729, EPI_ISL_64956, EPI_ISL_1081370, EPI_ISL_163493, EPI_ISL_173878).
