# Supplementary material for: A pan-influenza monoclonal antibody neutralizes H5 strains and prophylactically protects through intranasal administration
Source: Sci Rep. 2024 Feb 15;14:3818. doi: 10.1038/s41598-024-53049-5 (PMC10869794; doi:10.1038/s41598-024-53049-5)
Supplement: Supplementary file 1 — Supplementary Information. [file 41598_2024_53049_MOESM1_ESM.pdf]

SUPPLEMENTARY MATERIAL TO:

A pan-influenza monoclonal antibody neutralizes H5 strains and protects through intranasal administration

Anna L. Beukenhorst[1,2,3*], Jacopo Frallicciardi[2], Keira L. Rice[2], Martin H. Koldijk[2], Joana C. Moreira de Mello[2], Jaco M. Klap[2], Christoforos Hadjichrysanthou[4], Clarissa M. Koch[2], Kelly A.S. da Costa[5], Nigel Temperton[5], Babette A. de Jong[2], Helene Vietsch[2], Bertjan Ziere[2], Boris Julg[2], Wouter Koudstaal[2] & Jaap Goudsmit[2,6]

1. Department of Biostatistics, Harvard T.H. Chan School of Public Health, Boston, MA, USA
2. Leyden Laboratories BV, Leiden, The Netherlands.
3. Centre for Epidemiology, University of Manchester, Manchester Academic Health Science Centre, Manchester, UK
4. Department of Mathematics, University of Sussex, Brighton, UK
5. Viral Pseudotype Unit, Medway School of Pharmacy, University of Kent & University of Greenwich, Chatham, United Kingdom
6. Departments of Epidemiology, Immunology and Infectious Diseases, Harvard TH Chan School of Public Health, Boston, MA, USA.

Supplementary Table 1. Strains tested in the pseudotype virus neutralization assay (PNA).

Isolate Name	Isolate ID	Date	Region	Country	Host	Subtype	Clade
A/Anhui/1/2005	EPI_ISL_10058	2005-11-8	Asia	China	Human	A/H5N1	2.3.4
A/mallard duck/Netherlands/41/2015	EPI_ISL_328966	2015-9-15	Europe	Netherlands	Mallard	A/H5N1	EA_nonGsGD
A/Hong Kong/156/97	EPI_ISL_954	1997-XX-XX	Asia	Hong Kong (SAR)	Human	A/H5N1	0
A/Anhui/2021-00011/2020	EPI_ISL_1081370	2020-12-22	Asia	China	Human	A/H5N6	2.3.4.4h
A/Vietnam/1203/2004	EPI_ISL_10656749	2006-6-14	Asia	Vietnam	Human	A/H5N1	1
A/Indonesia/5/2005	EPI_ISL_121541	2005-XX-XX	Asia	Indonesia	Human	A/H5N1	2.1.3.2
A/Sichuan/26221/2014	EPI_ISL_163493	2014-4-21	Asia	China	Human	A/H5N6	2.3.4.4b
A/gyrfalcon/Washington/41088-6/2014	EPI_ISL_173878	2014-12-8	North America	United States	Gyrfalcon	A/H5N8	2.3.4.4c
A/whooper_swan/Mongolia/244/2005	EPI_ISL_64956	2005-XX-XX	Asia	Mongolia	Whooper swan	A/H5N1	2.2
A/chicken/Mexico/07/2007	EPI_ISL_244540	2007-XX-XX	North America	Mexico	Chicken	A/H5N2	Am_nonGsGD

Supplementary Table 2. Geometric mean HI titers in pooled sera one day before challenge (27 days after immunization). Lower limit of detection = 5

Mice	H1N1 (A/Victoria)	H3N2 (A/Cambodia)	B/Washington (Victoria lineage)	B/Phuket (Yamagata lineage)
Vaccinated	196	320	28	40
Unimmunized	≤ 5	≤ 5	≤ 5	≤ 5

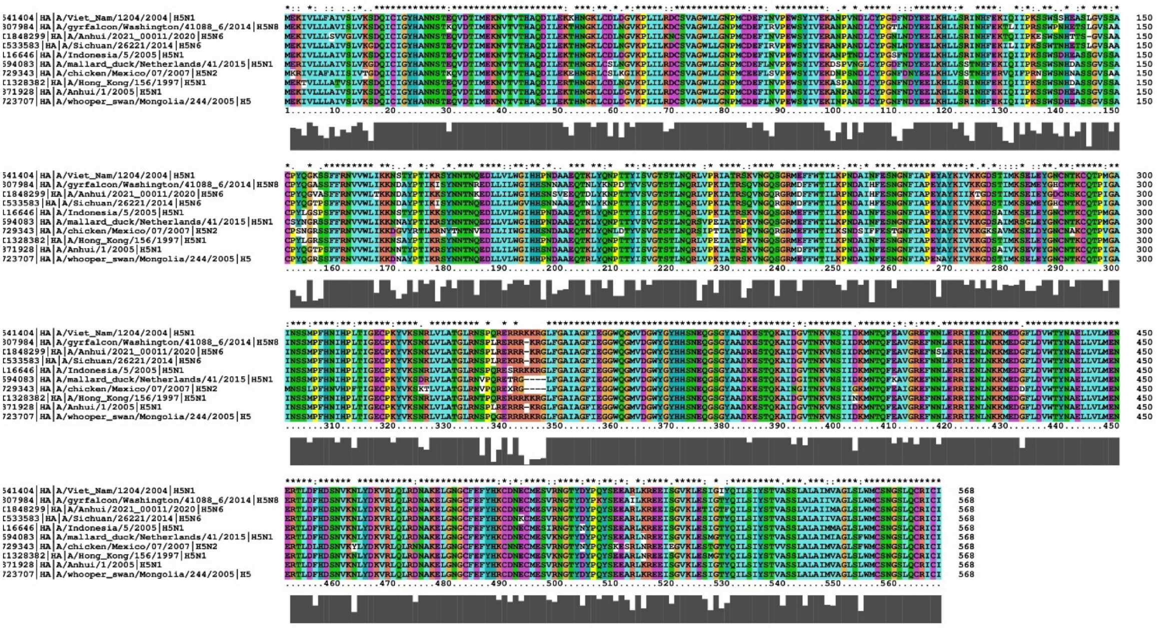

Supplementary Fig. 1. Alignment of the HA protein sequences for the A/H5 strains tested in the PNA.

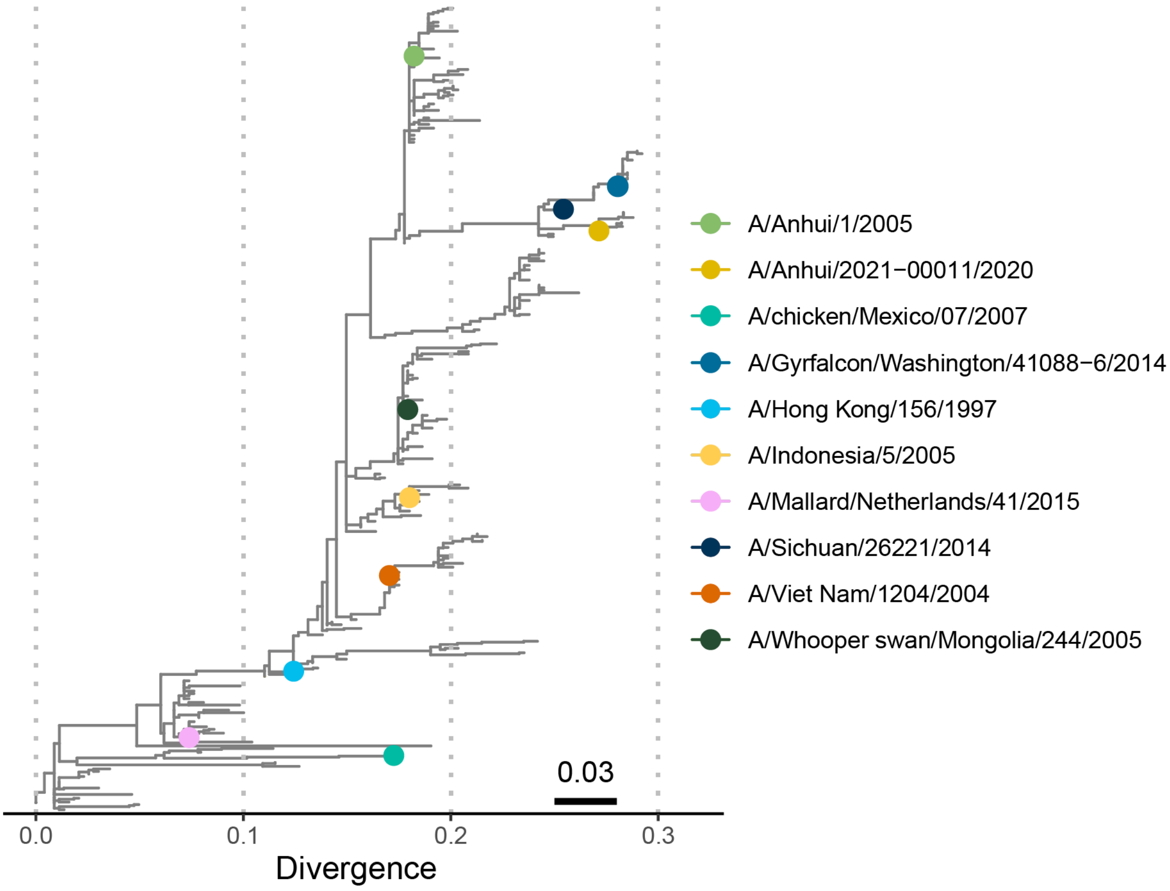

Supplementary Fig. 2. Complete phylogenetic tree based on the identity of the hemagglutinin protein sequence from 632 isolates. A/chicken/Scotland/1959 was used as a root for building the tree.
